# Proof-of-concept study: Homomorphically encrypted data can support real-time learning in personalized cancer medicine

**DOI:** 10.1186/s12911-019-0983-9

**Published:** 2019-12-04

**Authors:** Silvia Paddock, Hamed Abedtash, Jacqueline Zummo, Samuel Thomas

**Affiliations:** 1 Rose Li and Associates, Inc., 1101 Wootton Pkwy, Suite 400A, Rockville, MD 20852 USA; 2 Eli Lilly and Company, Lilly Corporate Center, Indianapolis, IN 46285 USA

**Keywords:** Homomorphic encryption, Learning system, Real-world evidence, Off-label treatment, Cancer

## Abstract

**Background:**

The successful introduction of homomorphic encryption (HE) in clinical research holds promise for improving acceptance of data-sharing protocols, increasing sample sizes, and accelerating learning from real-world data (RWD). A well-scoped use case for HE would pave the way for more widespread adoption in healthcare applications. Determining the efficacy of targeted cancer treatments used off-label for a variety of genetically defined conditions is an excellent candidate for introduction of HE-based learning systems because of a significant unmet need to share and combine confidential data, the use of relatively simple algorithms, and an opportunity to reach large numbers of willing study participants.

**Methods:**

We used published literature to estimate the numbers of patients who might be eligible to receive treatments approved for other indications based on molecular profiles. We then estimated the sample size and number of variables that would be required for a successful system to detect exceptional responses with sufficient power. We generated an appropriately sized, simulated dataset (*n* = 5000) and used an established HE algorithm to detect exceptional responses and calculate total drug exposure, while the data remained encrypted.

**Results:**

Our results demonstrated the feasibility of using an HE-based system to identify exceptional responders and perform calculations on patient data during a hypothetical 3-year study. Although homomorphically encrypted computations are time consuming, the required basic computations (i.e., addition) do not pose a critical bottleneck to the analysis.

**Conclusion:**

In this proof-of-concept study, based on simulated data, we demonstrate that identifying exceptional responders to targeted cancer treatments represents a valuable and feasible use case.

Past solutions to either completely anonymize data or restrict access through stringent data use agreements have limited the utility of abundant and valuable data. Because of its privacy protections, we believe that an HE-based learning system for real-world cancer treatment would entice thousands more patients to voluntarily contribute data through participation in research studies beyond the currently available secondary data populated from hospital electronic health records and administrative claims. Forming collaborations between technical experts, physicians, patient advocates, payers, and researchers, and testing the system on existing RWD are critical next steps to making HE-based learning a reality in healthcare.

## Background

### The complex nature of cancer and the increasing importance of targeted medicines

Cancer is a complex genetic disease, and researchers are now beginning to re-classify tumors based on their molecular composition rather than anatomical site [1]. Recently, the U.S. Food and Drug Administration (FDA) approved a treatment for the first time for tumors with damaged DNA repair mechanisms and therefore unusually large numbers of mutations, regardless of their anatomical origin [2]. Clinical trials of the future will likely incorporate more such molecular knowledge, which spans across anatomical sites.

**Fig. 1:**
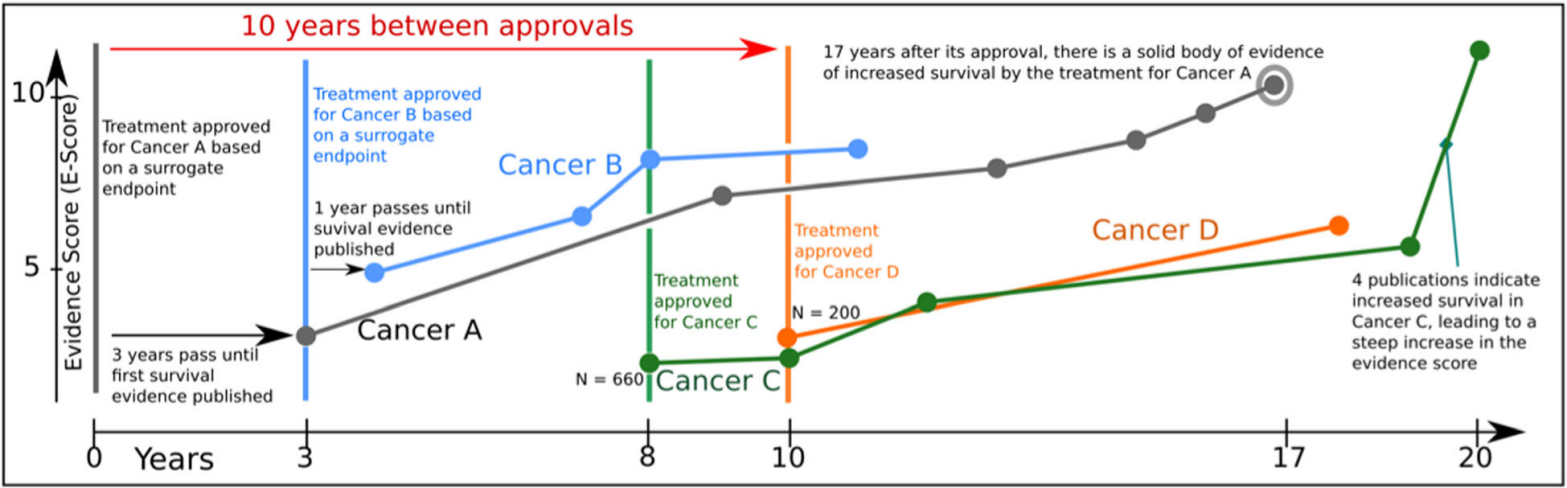
The complex evolution of anticancer treatment evidence and approvals. Adapted from the PACE CII online tool at http://scoringprogress.com. The x-axis shows time since the first approval; the y-axis shows the E-score, a measure of strength of evidence that the approved treatment increases overall survival. Vertical lines indicate the year during which the treatment was first approved for the respective cancer. Year 0 indicates the first approval of this treatment for any cancer

Today, however, most clinical trials are conducted by anatomical location, and drug approvals are granted accordingly, even though each anatomically defined cancer is heterogenous and includes only a subgroup of patients who respond. To understand which molecularly targeted treatments have demonstrated efficacy in multiple anatomical cancer types, we can use the PACE Continuous Innovation Indicators™ (CII). We previously developed the PACE CII as a free tool that allows researchers and advocates to track progress against 10 common solid tumors [3]. We published the methodology [4] and update the tool at least annually. The PACE CII allows deep analyses of approval pathways and molecular treatment classes. Figure 1 shows results from an example analysis of a treatment that has been approved for four cancers (Cancers A-D).

This analysis shows that the treatment was approved first for Cancer A (gray line) 3 years before published evidence demonstrated that the treatment improved overall survival. The same treatment was later approved for Cancers B, C, and D, with the latter approval occurring 10 years after the initial approval. For Cancers C and D, the approval coincided with publication of significant evidence for increased overall survival.

Although the proportion of patients who respond to treatment in Cancer D may be the same or even larger than those who respond in Cancer A, the lower prevalence of Cancer D may have contributed to the 10-year delay between the respective approvals, because it takes longer to recruit for and complete clinical trials for rare diseases. There may be additional cancer types that have never been tested and that harbor substantial numbers of patients that would be considered “exceptional responders” to this treatment. The field needs additional resources to find the patients with sensitive mutations in each anatomical group.

Several organizations have embarked on very different, molecularly classified studies. The American Society for Clinical Oncology (ASCO) has recently expanded its Targeted Agent and Profiling Utilization Registry (TAPUR) study—a nonrandomized trial of FDA-approved, molecularly targeted treatments—for several indications based on encouraging initial results [5, 6]. The National Cancer Institute (NCI) has funded several trials, including the “genotype to phenotype” Molecular Analysis for Therapy Choice (MATCH) study and the “exceptional responders” identification initiative [7]. Initial results from these trials show modest successes, indicating the need for even larger sample sizes to obtain robust statistical data on responses [8]. Finding one exceptional responder in a modest dataset of 40–50 patients may be a fluke, but finding 5% such responders in thousands of patients would reveal an interesting pattern for further investigation.

### Real-world problems requiring real-world evidence (RWE)

Studying patients treated in real-world practice settings, as opposed to those participating in clinical trials of investigational treatments, may play a key role in determining which treatments work best for which patients. Once a treatment is approved by the FDA for any indication, physicians may prescribe it to patients using professional judgment, including for unapproved, “off-label” indications. Indeed, as many as 71% of adult cancer patients may receive an off-label treatment [9]. With less than 5% of cancer patients participating in clinical trials [10], learning systematically from patients treated off-label in real-world practice settings will be a key to understanding how to more effectively use currently approved treatments.

Although most countries allow off-label prescribing, the rules are vague and tend to require a reasonable rationale to support the treatment [11, 12]. These ill-defined requirements generate a considerable additional workload for physicians, who often must generate comprehensive letters citing preclinical and clinical data to support insurance reimbursement for off-label use [13]. These efforts could be better directed toward participating in real-world learning systems, which could eventually lead to clearer reimbursement decisions.

Based on published data about off-label use even in the pre-personalized area, it is clear that we are not learning from considerable information about possible exceptional responders that are generated in daily practice [14]. In the example shown in Fig. 1, clinical trials with a total of 860 patients led to approval of a treatment for Cancers C and D that had already been approved for Cancer A. However, during the 10-year course of those trials, thousands more patients were treated off-label with this treatment in the United States alone, and their experiences did not feed back into the learning cycle in a systematic way.

### Privacy concerns, data ownership, and lack of trust as barriers to real-world learning

To overcome the learning gap in the real world, many researchers have aimed to use RWD more efficiently. However, they have encountered backlash amid concerns about multiple testing, lack of control over the data, ownership of the results, and privacy. In the past, researchers addressed these issues by (1) completely anonymizing and then publicly sharing data or (2) keeping the data private and allowing access only through strictly controlled data access agreements.

In the first case, the goal is to completely unlink the data from the patients, who sometimes were not required to provide informed consent, because the data were used for secondary purposes and could not be traced back. “Anonymizing” data while preserving its utility is a daunting task, mainly because of the presence of quasi-identifiers—elements other than direct identifiers (e.g., name, address) that alone or in combination with other data can be used for re-identification. Several notable examples of successful re-identification of “de-identified” data met with media outcry and increased distrust in data sharing and the research enterprise [15]. Aware of these risks, pharmaceutical companies submitting individual-level clinical trial data to regulatory agencies such as the FDA and the European Medicines Agency (EMA) usually conduct extensive steps to de-identify quasi-identifiers (e.g., visit dates), resulting in individual-level data of low utility, often containing little more information than the published group-level results [16].

In the second case, researchers working with the strictly controlled data have been required to complete increasingly detailed protocols before obtaining the data [17] to ensure that hypothesis-generating steps could be distinguished from hypothesis testing, and that the multiple testing problem was managed. These requirements have created a new barrier to analysis of RWD, because science evolves quickly, and a protocol that seemed relevant a year before the planned study may need multiple modifications once the actual data become available. Thus, when researchers obtain results about the actual number of cases overall and in different subgroups, they may have to modify their analysis plan, and it is not possible to tell whether they might have already seen the results yet. This creates issues of trust, as it is possible that revised analysis plans will be engineered to produce desired results.

### Homomorphic encryption as a possible accelerator for RWE insights

**Fig. 2:**
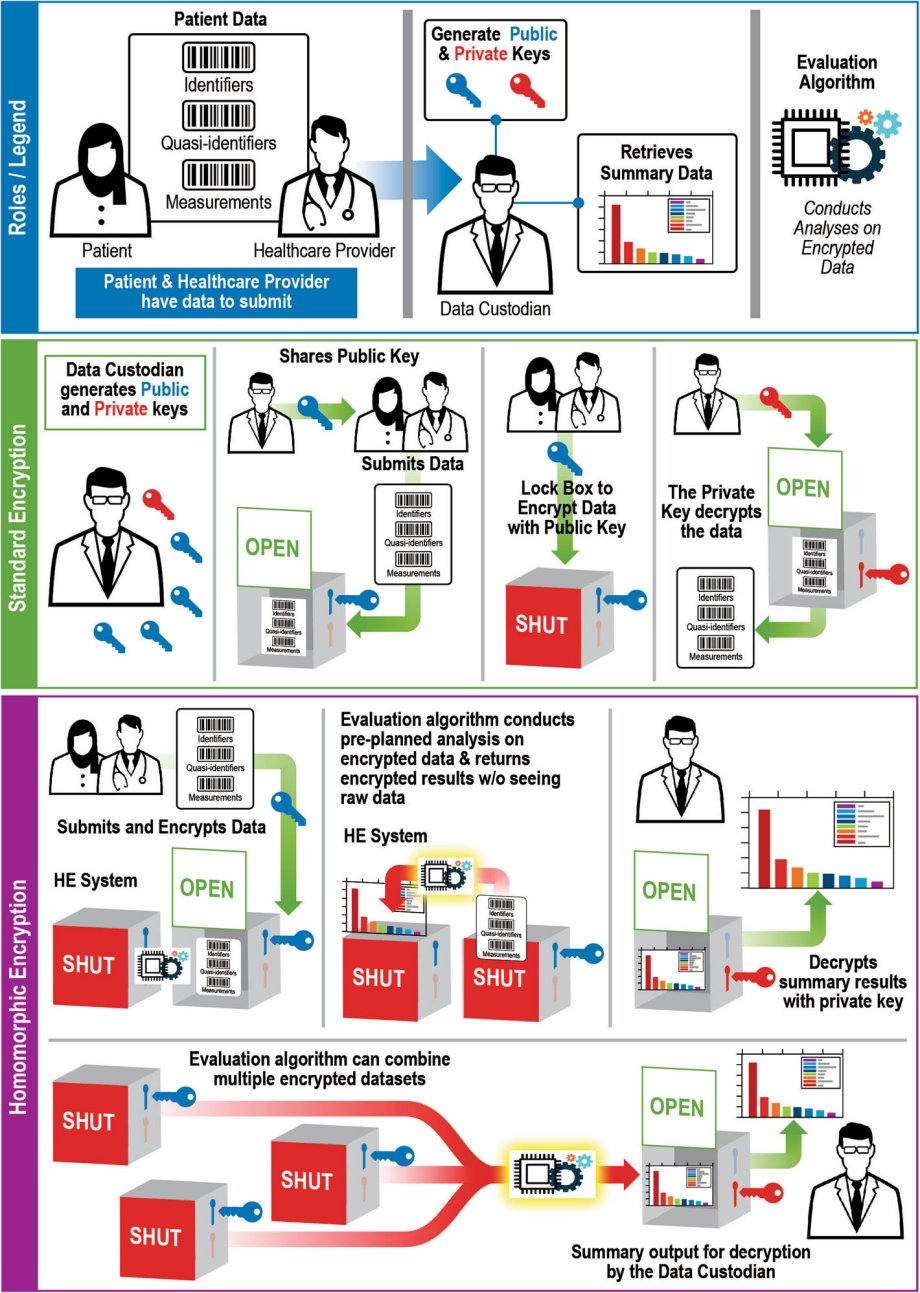
Schematic of performing computaitons on encrypted data using homomorphic encryption

**Fig. 3:**
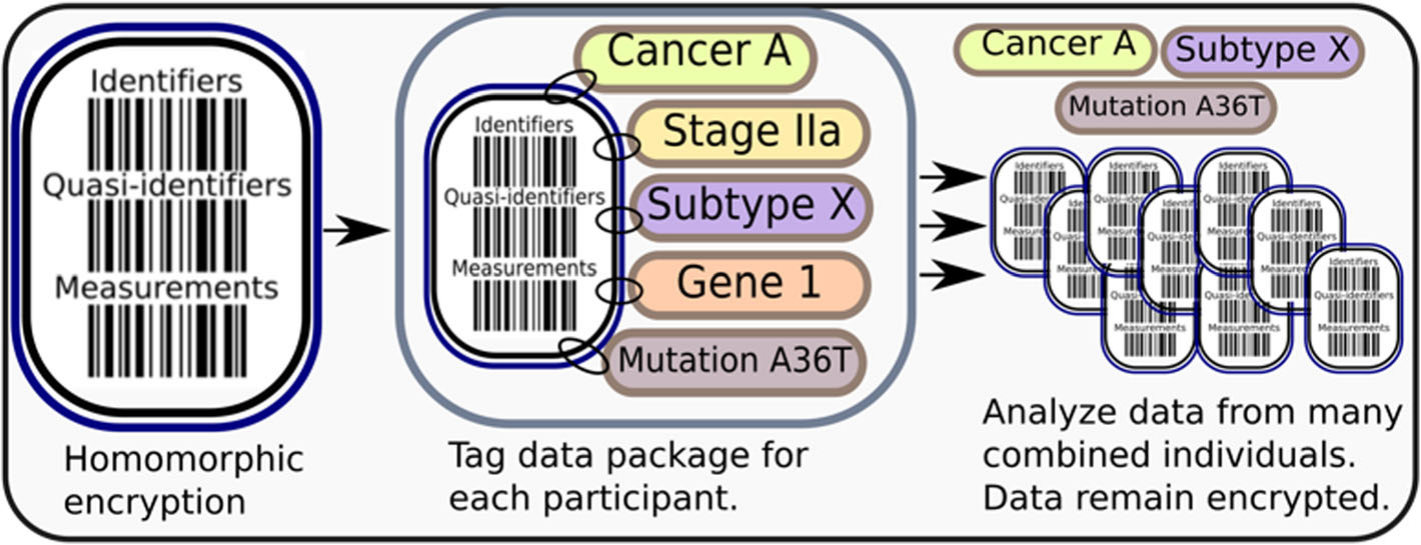
Data mart for personalized cancer treatments powered by homomorphic encryption

Homomorphic encryption allows for analysis of data while the data remain encrypted. A data mart that hosts the homomorphically encrypted data would perform the analyses and control the number of performed tests, with no knowledge about the raw data except its structure (Figs. 2 and 3) [18]. A systematic implementation of such a system could identify exceptional responders to treatments in very large datasets and could accelerate learning without jeopardizing patient privacy. An important feature of the system suggested herein is the combination of data “tags” (e.g., cancer type, mutated gene) that allow researchers to conduct power calculations and modify analysis plans as the data arrive. Because all patient-level data such as identifiers, visit dates, and measurements are encrypted homomorphically, none of this information is ever available to the analysts. This system thus balances the data confidentiality with the flexibility that rapidly evolving cancer science demands.

The goal of this proof-of-concept study was to examine the feasibility of using the current homomorphic encryption technology to identify exceptional cancer treatment responders from a simulated longitudinal dataset.

## Methods

### Estimation of the upper bound of numbers of individuals for each treatment

We used the PACE CII to identify treatment label expansions and other published literature to estimate the numbers of patients who might be eligible to receive treatments approved for other indications based on their molecular profiles. We estimated that the number of patients would be unlikely to exceed 5000 patients per treatment each year in the United States for the most frequent cancers (breast, prostate, lung, and colorectal), and remains at or below 1000 for the less frequent cancers (e.g., gastric, pancreatic, and liver). This estimate was based on PACE CII data for the most common treatment label expansions in the past (an average 9.3 years, data not shown), global incidence estimates from GLOBOCAN [19], data from the Surveillance, Epidemiology, and End Results Program (SEER) on stage distribution [20] and published data on off-label use (about 13% for breast cancer) [21, 22]. Because each of the cancers is heterogeneous (multiple different etiologies and genetic mutations), we conducted power analyses to ensure that samples of 1000 or 5000 patients would have enough power to detect differences between groups. We used the “pwr” package in R to perform these calculations [23]. We found that a moderate effect (effect size of 30%) would be detectable with the 1000 patients in a chi-squared test with 100 degrees of freedom at a significance level of *p* = 0.001 with 95% power. The larger sample of 5000 entries would allow detection of a smaller effect size, down to about 13%. This indicates that these target sample sizes would be sufficiently powered to detect moderate to small effects even when carrying out many parallel tests. Thus, our challenge was to show that we can learn from these patients through a secure and practical system. For the purpose of this study, we generated two datasets of 1000 and 5000 patients, and measured the time it took to finish the analyses.

### Data sources

While the goal of our inititative is to build a real-world learning system that can analyze individual patient data from a wide geographical catchment area (e.g., through smartphone apps linked to a central server nation-wide), a proof-of-principle study should be carried out on simulated data to avoid privacy issues and possible revelation of patient information while testing the homomorphic encryption procedures. We therefore decided to perform this study on simulated data that mirrored the challenges expected in the real world but did not contain any actual real-world patient information. The perl script generating the simulated dataset is provided in Additional file 1. In brief, the script generates random numbers based on desired distributions with a specified mean and standard deviation.

The simulated dataset had to fulfill two criteria to test the feasibility of homomorphic encryption for our purposes: (1) be able to add values across all patients in the dataset to count the number of exceptional responders in the dataset. For this challenge, we generated arrays of 1000 and 5000 values and measured the time it took for the algorithm to finish the calculation (code in Additional file 2); (2) the dataset should further contain a multiplicative task, such as determining the total drug exposure, to normalize values across patient records and combine them for further analyses (code in Additional file 3). For this purpose, we generated 1000 and 5000 records of simulated monthly patient weight information (in kg) and monthly drug dosage (in mg/kg) to determine the exposure for a given month [in mg]. As long as the computation time was less than the study time, we considered the analysis to be feasible, because it would not slow down an actual real-world study.

To create simulated data of the kind and size that would, eventually, be required for real-world analyses, we chose to simulate a dataset with 8 ± 1 months’ standard survival and 11 ± 1 months of exceptional survival. The total dataset was a combination of the standard and exceptional survivors, containing 5% exceptional survivors and 95% standard survivors.

**Fig. 4:**
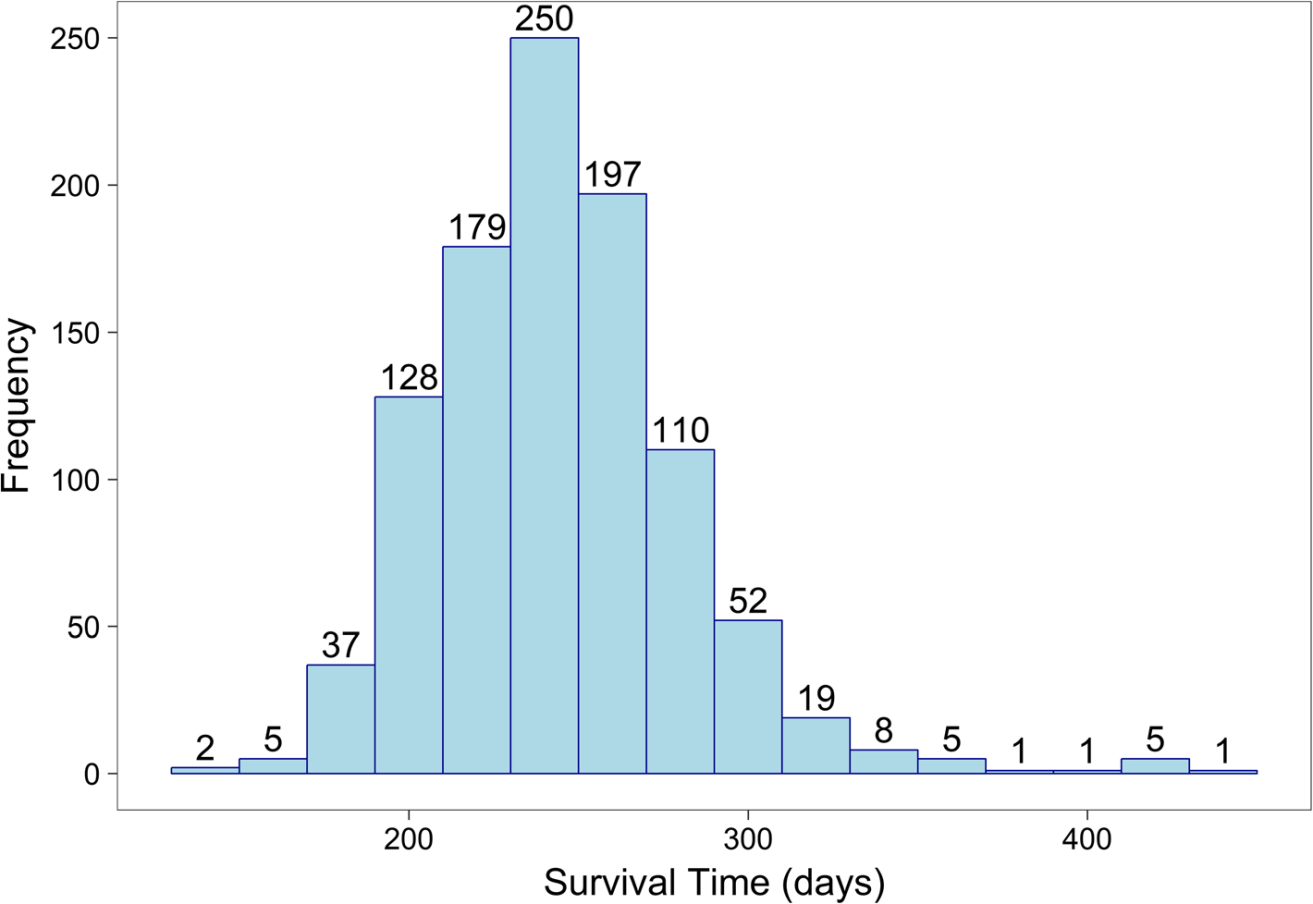
Distribution of survival times in the simulated dataset with 5% exceptional responders. Addition of the responders leads to an elevated tail of the distribution on the right side. The y-axis shows the number of simulated patients surviving during the time indicated on the x-axis

We generated simulated datasets based on the above requirement to capture basic information (e.g., a patient identifier) and data about treatment encounters (in our example, 48 monthly visits across the study period). For the exceptional responder identification, the data generation comprised two steps: we first generated patient response data over 48 months of our hypothetical study. We then used these data to create an array with flags (‘0’, or ‘1’) indicating for each patient if exceptional responder status has been achieved at this point. Figure 4 shows the survival distribution (in days) of the simulated cohort.

### Homomorphic encryption method and choice of parameters

We used an experimental form of the homomorphic encryption by Fan and Vercauteren (FV) [24] implemented in the ‘HomomorphicEncryption’ R package [25]. The package contains a command (‘parsHelp’) to select parameters based on the desired security level, maximum value that needs to be stored (default = 1000), and multiplicative depth. We base our analyses on parameters calculated by the helper function. A security-level of 128, for example, means that a brute force attack would need to try 2^128^ different combinations to find the correct key. Even with millions of very fast computers available, this effort would take billions of years.

One common feature of all encryption schemes (regular and homomorphic) is the need to add noise, so that a hostile agent cannot obtain one correct answer of the algorithm and from there derive the key. This noise grows with each manipulation of the data and grows faster with multiplications than with additions. The multiplicative depth parameter reveals information about the number of consecutive multiplications in the FV scheme that can be performed until the noise level becomes so large that the data get disrupted. The higher this parameter is chosen, the more complex computations can be performed on the data. We explored values up to a depth of 16 consecutive operations to allow multiple levels of computations to be performed on the data.

In the real world, new records are being added to patients’ electronic medical records periodically that need to be encrypted and submitted to data mart repositories for observational research. This requires the matching of an encrypted identifier from one dataset (e.g., the incoming data) with an identifier in another dataset (the data mart repository). Other researchers have addressed this problem by, for example, implementing a homomorphic method based on Bloom filters [26] and showing that the addition of the homomorphic encryption step does not diminish the accuracy of the matching of records. Assuming that a practical solution can be implemented based on existing strategies, we therefore excluded this step from the study.

Identification of exceptional responders mainly requires counting the numbers of patients alive at a given time point (addition), which introduces less noise than does multiplication. In brief, we assigned a random start date within 6 months to each patient, encrypted all patient records, and added the numbers of patients at each month that were labeled as “exceptional responders,” while these data remained encrypted. We measured the time until all exceptional responders were identified.

In a second step, we calculated the total drug exposure for each patient during the study period by multiplying the patient’s weight with the received dosage to demonstrate the feasibility of multiplicative operations.

## Results

The computation times from our simulations are summarized in Tables 1 and 2, which shows the expected trade-off between the level of security, the depth of possible calculations on the data (most of the actual manipulations will be addition), and time. All calculations finished within reasonable time frames on a desktop computer.

**Table 1:** Experimental computing time (seconds) for identification of exceptional responders using addition across records of all included patients

Number of patients	Lambda (security measure in bits)	Multipli-cation depth	Time to encrypt vector for one variable	Time to add encrypted vector for all patients	Addition time multiplied by 100 variables per analysis	Time to decrypt results^a^
1000	128	8	70.5	0.6	60	0.1
1000	256	8	142.7	1.2	120	0.2
1000	256	16	316.7	2.8	280	0.4
5000	128	8	234.2	1.8	180	0.1
5000	256	8	616.7	7.6	760	0.2
5000	256	16	1583.8	2512.2	25,120	0.8

^a^The final decryption times of the results occur only once at the end of each analysis. All times in seconds

**Table 2:** Experimental computing time (seconds) for identification of total drug exposure (multiplication)

Lambda (security measure in bits)	Multiplication depth	Time to encrypt all data for one patient	Time to multiply weight and dose data for all months	Time to add total exposure for one patient	Time to decrypt summary data for one patient	Total computation time for one patient	Estimated total time for 5000 patients
128	8	13.3	14.7	2.6	0.2	30.8	42 h
256	8	13.3	14.9	2.6	0.2	31.0	43 h
256	16	56.8	42.4	5.3	0.8	105.3	146 h

^a^The final decryption times of the results occur only once at the end of each analysis. All times in seconds unless otherwise specified

**Fig. 5:**
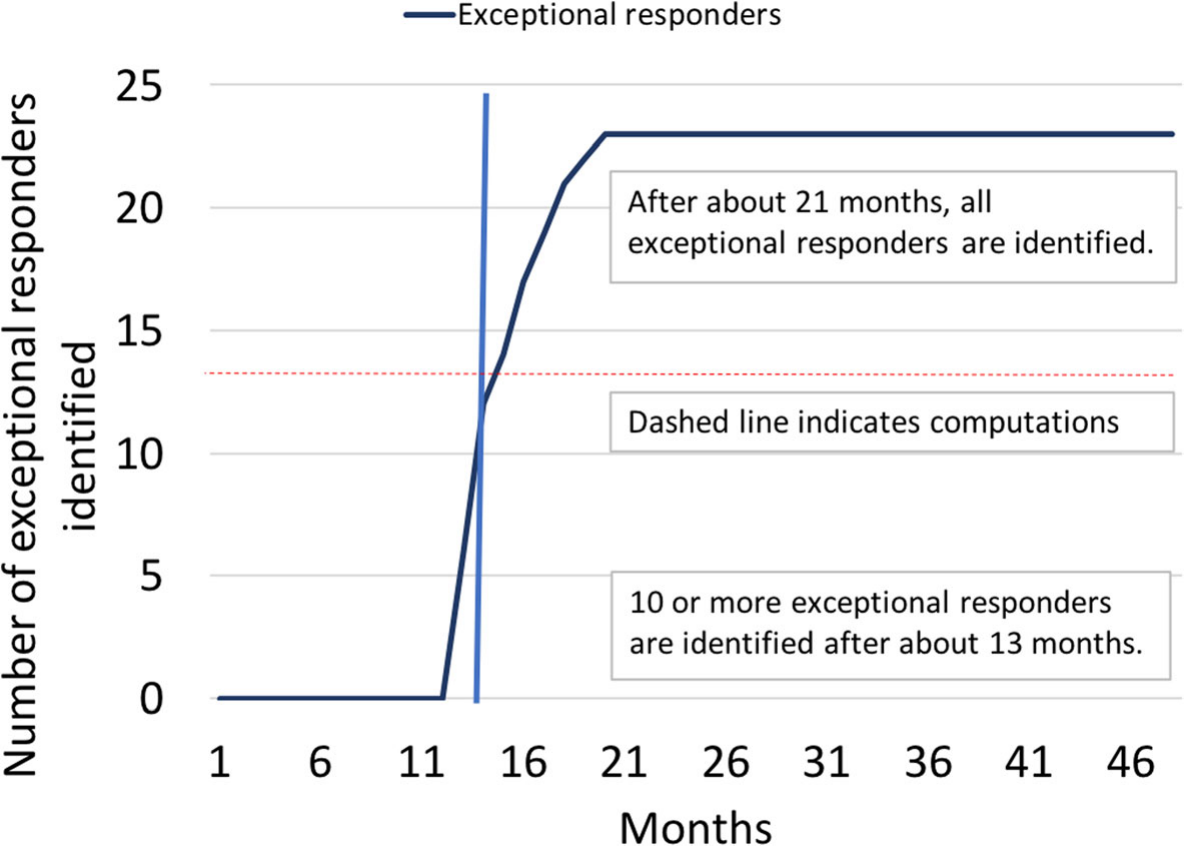
Identification of exceptional survivors in simulated dataset by homomorphic addition of encrypted records by month. The dashed red line shows the computations on the dataset. The computations can take several hours for each run, but they do not slow the pace of the study, because they occur in real time as the dataset grows and the study proceeds

The process of identifying exceptional responders is summarized in Fig. 5. Ten or more exceptional survivors are identified after about 13 months, and all exceptional survivors are identified after about 21 months. The computations to identify exceptional responders (i.e., adding the numbers of exceptional responders in the dataset) took at most 7 h per month (Table 1), which means that they did not slow the pace of the study. The rate-limiting factor of the study, therefore, was not the computation but the need to wait until enough exceptional survivors reach the pre-agreed survival duration threshold. We can fill this waiting period with computations on encrypted data (up to 24 h/day), in which case the computations would run continuously.

## Discussion

We herein show that analysis of data to identify exceptional responders based on homomorphic encryption is feasible even on a standard desktop computer. Of course, one would choose a more powerful system for real application, considerably expanding the possible depth and security of calculations. With HeLib [27, 28], PALISADE [29], TFHE [30] and SEAL [31], four powerful libraries are available for advanced users, with each package offering calculation optimization strategies such as batch-mode processing. The computation time estimates reported herein are thus to be considered an upper limit, the purpose of which is to show that computation duration would not be a bottleneck for the envisioned real-world study of exceptional responders.

The computation time for this study is considerably longer than that seen for standard analyses, which would occur after decryption of the submitted data. This agrees with findings from other groups, who have reported homomorphic encryption-based systems to run up to 40,000 times slower than regular computers [32, 33].

However, our proof-of-concept study shows that the increased computation time (days to weeks to finish complete sample sets) is negligible compared to the delays due to lack of learning from real-world experiences, which can last for decades, as shown in Fig. 1. If patients and their physicians know that the contributed health records data will never be decrypted, they will be more willing to participate in studies—leading to accelerated learning due to increased statistical power. Furthermore, computations can “tag along” in real time, further offsetting the additional time needed to compute encrypted data (red dashed line in Fig. 5).

Identification of the best solution for the current problem will be aided by the vibrancy of the field of homomorphic encryption. It is likely that solutions that are tailored to specific problems will begin to emerge. A practical, leveled, and somewhat homomorphic scheme is sufficient to identify exceptional responders to cancer treatments, and can be implemented with minor investments in technology.

Real-world collaborations between clinicians and programmers on defined problems are feasible and ready for prime time. Researchers in the homomorphic encryption community are actively looking for practical problems to solve. For example, during a summer 2017 workshop [34], one group identified genomics and the “match maker” as a possible application that could be implemented within 1 year’s time. For our proposed homomorphically encrypted learning system, no additional investment in technology would be required as physicians could submit the data via an App downloadable at the point of care. Larger scale efforts (e.g., genome-wide data analysis) remain beyond the scope of this system and may be better addressed by physical security solutions, as described by others [32, 33].

Payers would benefit from the refined understanding of which patients would benefit from which treatments—including those not currently approved for certain indications—that would be gained from adoption of a homomorphically encrypted learning system. In the long term, learning from such a system would help to improve outcomes of insured populations while minimizing waste on treatments understood to be less effective. Meanwhile, payers may be willing to accept participation in such a system as sufficient justification for reimbursement of off-label treatments, thereby reducing the reporting burdens on practicing physicians wishing to use treatments off-label based on molecular hypotheses. Other solutions appear feasible, such as managed-access agreements, through which the treatment’s manufacturer would be rewarded for positive responses only until the evidence for the benefit of the treatment solidifies.

During a recent workshop discussion, regulators expressed substantial hesitation to use real-world experiences for detection of small effects because of greater noise in large real-world datasets, but there is possible willingness to incorporate the data to determine label extensions [35]. The number of patients that we expect to be feasible to include are considerably higher than the number of patients included in randomized clinical trials or the current voluntarily patient-provided health data during the same period. The learning system described in this concept paper could be used to support such genomic profile-based extensions based on the knowledge of presence of consistently found exceptional responders across large datasets.

Our study has several limitations. First, our analysis is based on a research implementation of the algorithm, and a complete, practicable implementation would require additional steps of data validation and security testing. For example, in addition to the 48 variables tracking monthly response status during the study period, one would need to add a substantial number of additional variables for consistency checks (e.g., checksums to ensure that the correct records have been updated and that complete data integrity is maintained).

Second, we assume that patients and providers will appreciate the new technology. Although the technical capabilities for the identification of exceptional responders to targeted therapies clearly exist, patients must feel comfortable with having their results posted to a data mart. Similarly, providers must be assured of the data security and that the system will not disrupt clinical workflows. Therefore, an important next step is to conduct focus groups with patients and providers to explore how this new technology would be received and what level of security would be required to assure participating providers and patients that their privacy will be protected. Only when people clearly exhibit comfort with submitting their data to the system can we confirm that the system can lead to the envisioned manifold increase in high-utility RWD.

## Conclusions

Homomorphic encryption methods are receiving increased attention for all kinds of cloud-based applications [36]. Nonetheless, to our knowledge, there have been no practical implementations in the health care sector. Technical feasibility of scaling a clinical homomorphically encrypted learning system, an understanding of risks and benefits by participating patients and physicians, and willing parties to undertake the effort are needed to facilitate adoption. We believe that the use of homomorphically encrypted learning systems to identify exceptional responders to cancer treatments will become accepted by patients as the data owners and will increase sample sizes and thereby learning from real world experience.

Because the data are never de-identified, this system allows researchers to correct errors or withdraw voluntarily submitted data from the sample if they, for any reason, withdraw their consent, ameliorating concerns about data integrity and ownership seen in other efforts.

A near-term implementation in the cancer field, in which off-label use based on genomic profiles is reimbursed if data are deposited in the encrypted data mart, is an attractive way forward to provide patients with the best treatments for them while ameliorating concerns about lack of learning from outcomes of patients treated in routine clinical practice or uncontrolled multiple testing. Building a homomorphically encrypted learning system now to (mathematically) simplify the task of identifying exceptional responders also lays the foundation for future analyses that could lead to, for example, biomarker discovery and personalized treatment protocols.

It is important to note that homomorphic encryption is not a panacea for all privacy concerns. The major advantage is that patients can rest assured that their data will never be decrypted after submission. Nobody will be able to use the dates of their doctor’s visits or other indirect identifiers to trace back the submitted data. But this does not mean that any data would be safe to submit even to a homormorphically encrypted system. Because it is possible to identify individuals even from pooled samples of genomic data [37], summary allele frequencies obtained from homomorphic calculations would be sufficient to tell with reasonable certainty if a known DNA sample is represented in an encrypted collection. Thus, any existing de-identification problem applying to summary data would still be present in the homomorphic context. Incorporation of genomic or other data containing rare patterns would, therefore, require additional safeguarding steps (e.g., not report allele frequencies under 5%).

As outlined in this paper, the next steps are to establish collaborations among technical experts, physicians, patient advocates, payers, and researchers, and to ensure large-scale buy-in by patients whose data we can learn from “blindly.” Testing the technology on an existing real-world dataset would provide further assurance of feasibility and would help identify challenges to address before a complete rollout on newly collected data. Successful application of homomorphic encryption in this context could spur development of additional, increasingly ambitious efforts to learn from the wealth of existing untapped real-world health care data, both in oncology and for any other disease with a need for real-world learning. A real-world example is provided in the vignette below.

## Supplementary information

**Additional file 1. **Simulated Patient Data. A Word file with Perl code to generate simulated patient data (*n* = 1000 or *n* = 5000)

**Additional file 2.** HE Challenge 1. A Word file with R code for testing of homomorphic encryption times in challenge 1 (addition)

**Additional file 3.** HE Challenge 2. A Word file with R code for testing of homomorphic encryption times in challenge 2 (multiplication)

**Additional file 4.** Vignette: An example of an HE encryption system in action. A Word file with text for a boxed vignette that illustrates the use of the homomorphically encrypted data

## Data Availability

All computer code provided in supplemental files.

## Abbreviations

ASCO: American Society for Clinical Oncology
CII: Continuous Innovation Indicators
EMA: European Medicines Agency
FDA: Food and Drug Administration
FV: Fan and Vercauteren
HE: Homomorphic Encryption
MATCH: Molecular Analysis for Therapy Choice
NCI: National Cancer Institute
PACE: Patient Access to Cancer care Excellence
RWD: Real-World Data
RWE: Real-World Evidence
SEER: Surveillance, Epidemiology, and End Results Program
TAPUR: Targeted Agent and Profiling Utilization Registry
