# Supplementary material for: Proof-of-concept study: Homomorphically encrypted data can support real-time learning in personalized cancer medicine
Source: BMC Med Inform Decis Mak. 2019 Dec 4;19:255. doi: 10.1186/s12911-019-0983-9 (PMC6894133; doi:10.1186/s12911-019-0983-9)
Supplement: Supplementary file 1 — Additional file 1. Simulated Patient Data. A Word file with Perl code to generate simulated patient data (n = 1000 or n = 5000) [file 12911_2019_983_MOESM1_ESM.docx]

*#!/usr/bin/perl -w*

**use** strict; **use** Math::Random; **use** Math::Round;

*#generate normally distributed survival data for regular and exceptional survivors*

**my** @regularsurvivors = random_normal(950, 240, 30); *#replace first number by 4750 for n=5000 dataset*

**my** @intregularsurvivors = round(@regularsurvivors);

**my** @exceptionalsurvivors = random_normal(50, 330, 60); *#replace first number by 250 for n=5000 dataset*

**my** @intexceptionalsurvivors = round(@exceptionalsurvivors);

**my** @allPatients; **push** (@allPatients, @intregularsurvivors); **push** (@allPatients, @intexceptionalsurvivors);

*#generate random start dates for each participant*

**my** $minimum = 30; **my** $maximum = 180; **my** $ID = 1;

*#generate output datafiles*

**open** OUT, '>simpatdata1000.txt' **or** **die** "simpatdata1000.txt: $!";*#replace 1000 by 5000 for bigger dataset*

**open** WEIGHT, '>simpatweight1000.txt' **or** **die** "simpatweight1000.txt: $!";

**open** DOSE, '>simpatdose1000.txt' **or** **die** "simpatdose1000.txt: $!";

*#print headers for all output files*

**print** OUT "ID,startdate,survival,exceptiondate,m01,m02,m03,m04,m05,m06,m07,m08,m09,m10,m11,m12,m13,m14,m15,m16,m17,m18,m19,m20,m21,m22,m23,m24,m25,m26,m27,m28,m29,m30,m31,m32,m33,m34,m35,m36,m37,m38,m39,m40,m41,m42,m43,m44,m45,m46,m47,m48\n";

**print** WEIGHT "ID,startdate,survival,exceptiondate,m01,m02,m03,m04,m05,m06,m07,m08,m09,m10,m11,m12,m13,m14,m15,m16,m17,m18,m19,m20,m21,m22,m23,m24,m25,m26,m27,m28,m29,m30,m31,m32,m33,m34,m35,m36,m37,m38,m39,m40,m41,m42,m43,m44,m45,m46,m47,m48\n";

**print** DOSE "ID,startdate,survival,exceptiondate,m01,m02,m03,m04,m05,m06,m07,m08,m09,m10,m11,m12,m13,m14,m15,m16,m17,m18,m19,m20,m21,m22,m23,m24,m25,m26,m27,m28,m29,m30,m31,m32,m33,m34,m35,m36,m37,m38,m39,m40,m41,m42,m43,m44,m45,m46,m47,m48\n";

**foreach** **my** $patient (@allPatients){

**my** $randomstartdate = $minimum + **int**(**rand**($maximum - $minimum));

**my** $exceptiondate = $randomstartdate + 330;

**print** OUT "$ID,$randomstartdate,$patient,$exceptiondate";

**print** WEIGHT "$ID,$randomstartdate,$patient,$exceptiondate";

**print** DOSE "$ID,$randomstartdate,$patient,$exceptiondate";

**for** **my** $counter (1..48){

**my** $currentdate = $counter * 30;

**my** $currentdose = 0;

**if** ((($randomstartdate + $patient) > $exceptiondate) **and** ($currentdate > $exceptiondate)){**print** OUT ",1";**print** DOSE ",0";}

**else** {**print** OUT ",0";$currentdose = 200 * **int**(**rand**(4));**print** DOSE ",$currentdose";}

**my** $currentweight = 60 + **int** (**rand**(20));

**print** WEIGHT ",$currentweight";

}

**print** OUT "\n";

**print** WEIGHT "\n";

**print** DOSE "\n";

$ID++;}

**close** OUT;
