# Supplementary material for: Proof-of-concept study: Homomorphically encrypted data can support real-time learning in personalized cancer medicine
Source: BMC Med Inform Decis Mak. 2019 Dec 4;19:255. doi: 10.1186/s12911-019-0983-9 (PMC6894133; doi:10.1186/s12911-019-0983-9)
Supplement: Supplementary file 2 — Additional file 2. HE Challenge 1. A Word file with R code for testing of homomorphic encryption times in challenge 1 (addition) [file 12911_2019_983_MOESM2_ESM.docx]

library(tictoc)

library(HomomorphicEncryption)

p <- parsHelp("FandV", lambda=256, L=16)

k <- keygen(p)

simpatdata1000 <- read.table('simpatdata1000.txt', header = TRUE, sep = ",")

tic ("encoding")

encodedMonth20<-enc(k$pk, simpatdata1000$m20)

toc()

tic("addition")

encodedResult <- sum(encodedMonth20)

toc()

tic("decrypting results")

plainResult<-dec(k$sk, encodedResult)

toc()
