# Supplementary material for: Proof-of-concept study: Homomorphically encrypted data can support real-time learning in personalized cancer medicine
Source: BMC Med Inform Decis Mak. 2019 Dec 4;19:255. doi: 10.1186/s12911-019-0983-9 (PMC6894133; doi:10.1186/s12911-019-0983-9)
Supplement: Supplementary file 3 — Additional file 3. HE Challenge 2. A Word file with R code for testing of homomorphic encryption times in challenge 2 (multiplication) [file 12911_2019_983_MOESM3_ESM.docx]

*#load libraries*

library(tictoc)

library(HomomorphicEncryption)

*# load data*

sampleData <- read.csv("simpatdata1.csv")

totalExposure <- 0

*# set parameters*

p <- parsHelp("FandV", lambda=128, max=1000000, L=8)

k <- keygen(p)

*# encrypt datasets*

tic("encrypt data")

encDoseData <- enc(k$pk, sampleData$dose)

encWeightData <- enc(k$pk, sampleData$weight)

encTotalExposure <- enc(k$pk, totalExposure)

toc()

*# multiply vectors*

tic("multiply vectors")

encResultsData <- encDoseData * encWeightData

toc()

*# add all months*

tic("add all months")

for(i in 1:48){

encTotalExposure <- encResultsData[i] + encTotalExposure

}

toc()

*# decrypt result*

tic("decrypt result")

totalExposure <- dec(k$sk, encTotalExposure)

toc()

*## encoded with 256 bit security, 8 multiplicative depth*

*# clear workspace*

rm(list=ls())

*# load data*

sampleData <- read.csv("simpatdata1.csv")

totalExposure <- 0

*# set parameters*

p <- parsHelp("FandV", lambda=256, max=1000000, L=8)

k <- keygen(p)

*# encrypt datasets*

tic("encrypt data")

encDoseData <- enc(k$pk, sampleData$dose)

encWeightData <- enc(k$pk, sampleData$weight)

encTotalExposure <- enc(k$pk, totalExposure)

toc()

*# multiply vectors*

tic("multiply vectors")

encResultsData <- encDoseData * encWeightData

toc()

*# add all months*

tic("add all months")

for(i in 1:48){

encTotalExposure <- encResultsData[i] + encTotalExposure

}

toc()

*# decrypt result*

tic("decrypt result")

totalExposure <- dec(k$sk, encTotalExposure)

toc()
