# Supplementary material for: Proof-of-concept study: Homomorphically encrypted data can support real-time learning in personalized cancer medicine
Source: BMC Med Inform Decis Mak. 2019 Dec 4;19:255. doi: 10.1186/s12911-019-0983-9 (PMC6894133; doi:10.1186/s12911-019-0983-9)
Supplement: Supplementary file 4 — Additional file 4. Vignette: An example of an HE encryption system in action. A Word file with text for a boxed vignette that illustrates the use of the homomorphically encrypted data [file 12911_2019_983_MOESM4_ESM.docx]

**Vignette: An example of an HE encryption system in action.** Mr. Smith, a 45-year old never-smoker, is diagnosed with lung cancer. A sample taken from the tumor reveals a genetic mutation known to cause colorectal cancer, and a treatment targeting mutations in the affected gene has been approved for colorectal cancer but not for lung cancer. Mr. Smith’s doctor would need to write a justification to get the “off-label” treatment reimbursed, but because he participates in a real-world learning system, Mr. Smith instead installs an App on his smartphone that allows him to participate in this program. Mr. Smith enters basic demographic information and the app encrypts his data. He then sends the encrypted data to the learning system. The encrypted results of the study along with Mr. Smith’s historical clinical information get transmitted from the electronic health records (EHR) database by the care provider to the data guardian (e.g., a consortium of regulators and insurance companies), which is able to decrypt the results, but the users of this central data repository do not have access to any patient-level information. Because Mr. Smith knows that his data remain encrypted at all times, he feels confident to participate in the study through his smartphone. Only very few patients across the country have this type of mutation in their lung cancer disease, but because they can participate where they live through their smartphones, the system can track results from hundreds of matched participants. Based on his diagnosis, the system sends him messages in regular intervals asking him about his health status. The additional data that Mr. Smith reports are, again, encrypted before they get shared with the central database. If the targeted treatment works better than the standard treatment, Mr. Smith’s shared information, together with those from other patients around the country would lead to a significant breakthrough in discovering new therapies. The evidence to support the effectiveness of the new treatment would be generated through a comparative analysis on the server using the encrypted data to ensure that privacy of patients is secured during the process. Upon the approval of the generated RWE, the participating insurers reimburse the treatments of the participants in the learning system and obtain the benefit of faster real-world learning about novel treatments.
